# [Retracted] MicroRNA‑126 exerts antitumor functions in ovarian cancer by targeting EGFL7 and affecting epithelial‑to‑mesenchymal transition and ERK/MAPK signaling pathway

**DOI:** 10.3892/ol.2024.14548

**Published:** 2024-07-01

**Authors:** Yuhua Zhang, Xiaobo Qin, Juan Jiang, Wenjie Zhao

Oncol Lett 20: 1327-1335, 2020; DOI: 10.3892/ol.2020.11687

Following the publication of this paper, it was drawn to the Editor's attention by a concerned reader that certain of the Transwell migration and invasion assay data shown in Fig. 2B and D on p. 1332 had already appeared in previously published articles written by different authors at different research institutes, one of which has been retracted. Owing to the fact that the contentious data in the above article had already been published prior to its submission to *Oncology Letters*, the Editor has decided that this paper should be retracted from the Journal. The authors were asked for an explanation to account for these concerns, but the Editorial Office did not receive a reply. The Editor apologizes to the readership for any inconvenience caused.

